# Biomarkers of food intake for *Allium* vegetables

**DOI:** 10.1186/s12263-018-0624-4

**Published:** 2018-12-27

**Authors:** Giulia Praticò, Qian Gao, Claudine Manach, Lars O. Dragsted

**Affiliations:** 1 Department of Nutrition, Exercise and Sports, University of Copenhagen, Copenhagen, Denmark; 2 Department of Food Science, University of Copenhagen, Copenhagen, Denmark; 3 INRA, Human Nutrition Unit, Université Clermont Auvergne, F63000 INRA, Clermont-Ferrand, France

**Keywords:** *Allium* vegetables, Garlic, Onion, Shallot, Leek, Chives, Ramsons, Biomarkers, Intake

## Abstract

*Allium* vegetables are widely consumed around the world and are known for their potential bioactive components improving human health. These effects have been extensively investigated; however, the results were inconsistent in human studies. Biomarkers of food intake (BFIs) could provide objective measurements of food intake in observational studies and assess compliance in intervention studies. Therefore, the discovery and application of BFIs for *Allium* vegetables would facilitate the exploring and understanding of the health benefit of *Allium* vegetables. In this manuscript, we reviewed the currently used and potential candidate BFIs for *Allium* vegetables and evaluated their levels of validation. *S*-Allylmercapturic acid (ALMA), allyl methyl sulfide (AMS), allyl methyl sulfoxide (AMSO), allyl methyl sulfone (AMSO_2_), and *S*-allylcysteine (SAC), which are derived from organosulfur compounds, were shown to be promising candidate BFIs for garlic consumption. Further validation is needed to assess their robustness and concordance with other measures. Their applicability for the whole food group should be evaluated as well. *N*-Acetyl-*S*-(2-carboxypropyl)cysteine (CPMA) was detected in high levels in urine after both garlic and onion intake, suggesting that it may be used for the assessment of intake of *Allium* food group. The available information regarding its kinetics, robustness, and analytical performance is limited and needs to be assessed in further studies. No candidate BFIs specific to intake of onion, leek, chives, shallots, or ramsons were found. Untargeted metabolomics studies and further validation studies should be performed to discover more reliable BFIs for individual *Allium* vegetables and the whole food group. This paper serves as an example of Biomarker of Food Intake Reviews (BFIRev) and biomarker of food intake validation procedures.

**Electronic supplementary material:**

The online version of this article (10.1186/s12263-018-0624-4) contains supplementary material, which is available to authorized users.

## Background

### Introduction

*Allium* vegetables, onions and garlic in particular, constitute a part of the daily diet for most of the world’s population. They have also been extensively investigated for their potential health-promoting effects. Beneficial effects of garlic on blood pressure and blood lipids seem to be likely based on several recent human meta-analyses [1–5]. Garlic (*Allium sativum*) and onion (*Allium cepa*) extracts have also been proposed to positively modulate inflammation, cardiovascular diseases, and cancer [6, 7]. Other potential biological properties including antimicrobial, antioxidant, antiasthmatic, immunomodulatory, and prebiotic activities have also been reported [6]. However, none of these effects caused by intake of *Allium* or any of their constituents has, so far, been firmly documented in human trials. Any health benefits from *Allium* intake could be caused by a variety of constituents, such as the organosulfur compounds [6, 8], several flavonoids [9], saponins [10], and soluble fibers, including fructo-oligosaccharides (FOS).

In order to better elucidate the true potential of these food plants in relation to human health, it is important to assess intake accurately. Currently, the measurement of food consumption in observational studies largely relies on dietary assessment instruments such as food frequency questionnaire, food diary, etc., which are prone to systematic errors and recall bias. *Allium* vegetables are typically used in mixed dishes, which can make the self-evaluation of consumption particularly problematic when the consumer did not cook the dishes himself. Even if food consumption is controlled in intervention studies, compliance may be a problem when participants are not constantly monitored, which is rarely possible. Low compliance as well as imprecise reporting of food intake causes loss of power to find the true associations between food intake and disease. Compared to the currently applied dietary assessment instruments, biomarkers of food intake (BFIs) represent more objective measurements, ideally independent of external factors such as recipe or cooking process and of intrinsic factors such as memory. Therefore, BFIs are promising tools to provide objective measurement in observational studies and to assess compliance in intervention studies [11, 12]. The discovery and application of BFIs for *Allium* vegetables would help to better explore their potential health benefit. The objectives of this review were as follows: (1) summarize the actual knowledge related to candidate or currently used BFIs for *Allium* vegetable consumption, (2) provide an overview of the current level of validation of candidate BFIs, and (3) illustrate the use of Biomarker of Food Intake Reviews (BFIRev) [13] and biomarker of food intake validation procedures [14].

## Methods

The reviewing process was performed following the guidelines for biomarker of food intake reviews (BFIRev) [13].

### Selection of food groups

The *Allium* genus includes hundreds of species, both wild and cultivated as vegetables or ornamentals. In the vegetable group, the frequently consumed species—onion (*Allium cepa var. cepa*), garlic (*Allium sativum*), leek (*Allium ampeloprasum*), chives (*Allium schoenoprasum*), shallots (*Allium cepa var. aggregatum* and *Allium stipitatum*), ramsons (*Allium ursinum*), and garlic chives (*Allium tuberosum*)—were selected as representative foods in this group for further search. Although varieties of each species exist, there are only a few studies reporting the difference between them from a nutritional point of view. Therefore, the detail of the varieties has not been taken into consideration in the present review. Overview of compounds reported to be present in *Allium* vegetables was provided by food-related reference databases, FooDB (www.foodb.ca), Phenol Explorer (phenol-explorer.eu), and PubChem (www.ncbi.nlm.nih.gov), as well as references found in the biomarker literature search (see below).

### Search for relevant research papers on biomarkers of *Allium* intake

**Fig. 1:**
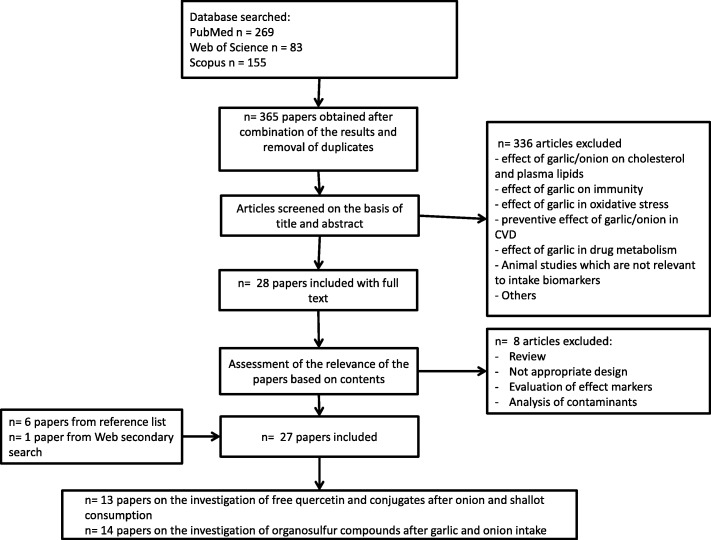
Flow diagram of study selection according to the BFIRev method [13]

Original research papers and reviews were searched in three databases (PubMed, Scopus, and the ISI Web of Knowledge) using combinations of the grouped search terms: (biomarker* OR marker* OR metabolite* OR biokinetics OR biotransformation) AND (trial OR experiment OR study OR intervention) AND (human* OR men OR women OR patient* OR volunteer* OR participant*) AND (urine OR plasma OR serum OR blood OR excretion) AND (intake OR meal OR diet OR ingestion OR consumption OR eating OR administration) AND (onion OR garlic OR leek OR *Allium* OR chives OR shallots OR ramsons). The fields used as a default for each of the databases were [All Fields] for PubMed, [Article Title/ Abstract/ Keywords] for Scopus, and [Topic] for ISI Web of Science. The research was carried out in December 2017 and was limited to papers in the English language, while no restrictions were applied for the publication dates. The research papers identifying or using potential biomarkers of intake for *Allium* vegetables were selected according to the process outlined in Fig. 1. Additional papers were identified from reference lists in these papers and from reviews or book chapters identified through the literature search. Exclusion criteria for the primary search were as follows: effect of *Allium* vegetables on cholesterol and plasma lipids, immunity, oxidative stress, and impact on cardiovascular diseases; effect of garlic in drug pharmacokinetics; animal studies which are not relevant to intake biomarkers; inappropriate study designs (e.g., confounded by other food groups); investigation of effect biomarkers; or analysis of contaminants.

### Identification of candidate BFIs

For each potential biomarker identified, a second search step was performed to evaluate the specificity of potential candidate BFIs. The search was conducted with (“the name and synonyms of the compound” OR “the name and synonyms of any parent compound”) AND (biomarker* OR marker* OR metabolite* OR biokinetics OR biotransformation) in order to identify other potential foods containing the biomarker or its precursor. In this second step, Scifinder and Google Scholar were also used as search platforms, in addition to the databases listed above. This second search was used to evaluate the apparent specificity of the initially identified BFIs. Based on an evaluation of biomarker specificity to *Allium* (see below), only the most plausible candidate BFIs have been reported in Table 1, including the information related to study designs and analytical methods. The reasons for inclusion or exclusion of BFIs from Table 1 have been reported in Table 2. These tables have been reviewed and agreed upon by all authors, and no additionally suggested biomarkers were found in the literature.

**Table 1:** List of studies reporting candidate biomarkers for *Allium* vegetable consumption

Food items	Study design	Number of subjects	Analytical method	Biospecimen	Candidate biomarkers	Primary reference(s)
Garlic Onion	Human single meal study Human single meal study	NA	GC-MS	Urine (24 h)	*N*-acetyl-*S*- (2-carboxypropyl) cysteine (CPMA) *S*-allylmercapturic acid (ALMA) *N*-acetyl-*S*- (2-carboxypropyl) cysteine (CPMA) *S*-allylmercapturic acid (ALMA)*	[19]
Garlic	Human single meal study	6	GC-MS	Urine (24 h)	*S*-allylmercapturic acid (ALMA)	[21]
Garlic	Placebo-controlled intervention study Cross-sectional study of Finnish vegans and controls	101 20	GC-MS	Urine (24 h) Urine (24 h)	*S*-allylmercapturic acid (ALMA) *S*-allylmercapturic acid (ALMA)	[22]
Fresh garlic	Randomized controlled parallel trial	NA	GC MS/MS	Urine (24 h)	*S*-allylmercapturic acid (ALMA)	[20]
Aged garlic extract	Human single meal study	1	HPLC-MS	Blood plasma Breath	*S*-allyl cysteine (SAC) Allyl methyl sulfide (AMS)	[18]
Fresh garlic	Human single meal study	7	GC-MS	Breath	Allyl methyl sulfide (AMS)	[28]
Fresh garlic	Human single meal study	1	GC-MS	Breath	Allyl methyl sulfide (AMS)	[30]
Garlic	Human single meal study	6	GC-MS	Breath	Allyl methyl sulfide (AMS)	[31]
Raw garlic	Human single meal study	1	PTR-MS	Breath	Allyl methyl sulfide (AMS)	[29]
Raw garlic	Human single meal study	12	GC-MS/O	Urine (24 h)	Allyl methyl sulfide (AMS)	[33]
			HRGC-MS		Allyl methyl sulfoxide (AMSO) Allyl methyl sulfone (AMSO_2_)	

*Abbreviation*: *PTR-MS* protontransfer-reaction mass spectrometry, *GC-MS/O* gas chromatography-mass spectrometry/olfactometry, *HRGC-MS* high resolution gas chromatography-mass spectrometry, *NA* not available

*No data is shown

**Table 2:** Summary of the selected candidate BFIs of *Allium* vegetables and the excluded biomarkers and reasons for inclusion or exclusion

Food item	Metabolites	Biofluid locations	Reason for inclusion or exclusion	Selected for the systematic validation as BFI	References
Garlic	ALMA	Urine	Specificity, suitable post-prandial kinetics, and dose-response	Yes	[19–22]
	AMS	Breath	Specificity, suitable post-prandial kinetics, and dose-response	Yes	[18, 28–31]
	AMS	Urine	Specificity, suitable post-prandial kinetics, and dose-response	Yes	[33]
	AMS	Breast milk	Uncommon sampling	No	[34]
	AMSO	Urine	Specificity, suitable post-prandial kinetics	Yes	[33]
	AMSO_2_	Urine	Specificity, suitable post-prandial kinetics	Yes	[33]
	SAC	Plasma	Probably specific, need for further investigation	Yes	[18]
	DADS	Urine	Too low concentration, scarce information on kinetics	No	[27]
	DADS	Breath	Too low concentration, too short half-life	No	[18, 29, 30, 32]
	DAS	Urine	Too low concentration, scarce information on kinetics	No	[27]
	DAS	Breath	Too low concentration, too short half-life	No	[18, 29, 30]
	DMS	Breath	Too low concentration, too short half-life	No	[29]
	DMDS	Breath	Unspecific, too low concentration	No	[30]
	DMDS	Urine	Unspecific, too low concentration	No	[27, 33]
	DMTS	Urine	Unspecific, too low concentration	No	[27]
	Acetone	Breath	Unspecific, too variable background	No	[28, 29]
	Organo-selenium compounds	Breath	Too low concentration	No	[30]
	Methanethiol	Breath	Too low concentration, too short half-life	No	[31]
	Allyl mercaptan	Breath	Too low concentration, too short half-life	No	[31, 32]
	AMDS	Breath	Too low concentration, too short half-life	No	[29–31]
	AMDS	Urine	Only detectable after the consumption of a high dose (30 g garlic)	No	[33]
	ADS	Breath	Too low concentration, too short half-life	No	[31]
	DATS	Breath	Too low concentration, too short half-life	No	[29, 30]
	Hexahydrohippuric acid	Urine	Unspecific	No	[19]
	2-propenethiol	Breath	Too low concentration, too short half-life	No	[30]
Onion	*N*-acetyl-*S*-(1Z)-propenyl-cysteine- sulfoxide	Urine	Probably specific, identification level II, need for further investigation	No	[48]
	Quercetin	Plasma/urine	Unspecific	No	[40, 42, 43, 56–61]
	Quercetin-3′-sulphate	Plasma/urine	Unspecific	No	[41]
	Quercetin-3-glucuronide	Plasma/urine	Unspecific	No	[41]
	Quercetin-4′-glucuronide	Plasma/urine	Unspecific	No	[47]
	Quercetin diglucuronide	Plasma/urine	Unspecific	No	[41]
	Isorhamnetin	Plasma/urine	Unspecific	No	[40, 43, 56, 59, 61]
	Isorhamnetin-3-glucuronide	Plasma/urine	Unspecific	No	[41]
	Isorhamnetin-4′-glucuronide	Plasma/urine	Unspecific	No	[47]
	Tamarixetin	Plasma/urine	Unspecific	No	[59, 61]
	Kaempferol	Plasma/urine	Unspecific	No	[56, 57]
	Flavonol metabolites	Plasma/urine	Unspecific	No	[40, 43, 46]
	Dimethyl sulfone	Urine*	Unspecific	No	[49]
	3-hydroxyphenylacetic acid	Urine*	Unspecific	No	[49]
Onion and garlic	CPMA	Urine	Probably specific, needs further investigation	Yes	[19]
Shallot	Quercetin	Plasma	Unspecific	No	[54]

*Abbreviation: ALMA S*-allylmercapturic acid, *AMS* allyl methyl sulfide, *AMSO* allyl methyl sulfoxide, *AMSO*_*2*_ allyl methyl sulfone, *CPMA N*-acetyl-*S*-(2-carboxypropyl)cysteine, *SAC S*-allylcysteine, *DADS* diallyl disulfide, *DAS* diallyl sulfide, *DMDS* dimethyl disulfide, *AM* allyl mercaptan, *AMDS* allyl methyl disulfide, *ADS* allyl disulfide, *DATS* diallyl trisulfide, *DMS* dimethyl sulfide, *DMTS* dimethyl trisulfide

*Data from animal study

### Application of validation criteria

To evaluate the current status of validation of candidate BFIs and to suggest the additional steps that are needed to reach the full validation, a set of validation criteria [14] was applied on each candidate BFI reported in Table 1. The assessment was performed by answering eight questions (Additional file 1: Text S1) related to the analytical and biological aspects of the validation together with a comment indicating the conditions under which the BFI is valid. The overview of the current levels of validation of candidate BFIs has been reported in Fig. 3.

## Results

Apart from common nutrients, the food databases point to the presence of a number of constituents in *Allium* vegetables that may form the basis for specific BFIs. In particular, organosulfur compounds are characteristic of the *Allium* species. These compounds include *S*-alk(en)yl-L-cysteine sulphoxides such as alliin (*S*-allyl-L-cysteine sulfoxide) in garlic. Also *N*-hydroxypyrithione derivatives have been described as present in the Persian shallot (*Allium stipitatum*) [15]. The presence of enzymes such as alliinase (alliin lyase, EC 4.4.1.4) that cleave the alkylsulphoxides upon crushing or cutting of the vegetables produces reactive species leading to the formation of aminoacrylate along with a large number of dialkyl di- and polysulphides and their oxidation products as well as sulphenic and sulphonic acids, and alkyl sulphoxides, including the lachrymatory factor, propanethial *S*-oxide. The most widely studied of these may be the garlic degradation product, allicin, S-Prop-2-en-1-yl-prop-2-ene-1-sulfinothioate, a thiosulfinate. Heat treatment inactivates the enzymes but not the alkylsulphoxides [16] so that all of these compounds, their enzymatic degradation products, and their human and gut microbial metabolites may form the basis for BFIs. Another group of constituents commonly found in *Allium* are polyphenols, especially flavonols such as quercetin and kaempferol glycosides, and anthocyanins in the red varieties and in some of the edible flowers. *Allium* anthocyanins include delphinidin and cyanidin glycosides and some more complex anthocyanin colors found in the purple flowers of, for example, chives. While onion has a particularly high content of quercetin glucosides, no polyphenols specific only to any of the *Allium* vegetables were identified in the databases. *Allium* vegetables may also contain several monoterpenoids, lignans, and other groups of compounds as inferred by genetic analysis; however, little information is available on the actual presence of these compounds. The *Allium* vegetables are also rich in soluble fibers of the fructan type that are resistant to human metabolism and substrates for the gut microbiota. Fructans are found also in several other vegetables, including Jerusalem artichoke, and are therefore not specific to *Allium*.

**Fig. 2:**
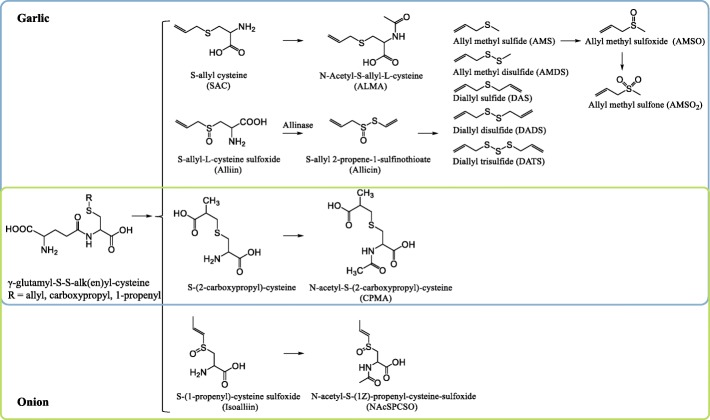
Organosulfur compounds in *Allium* vegetables and their metabolites in humans

The search process for BFIs identified 507 papers, which were reduced to 365 after the removal of duplicates. Subsequent screening of abstracts and titles reduced the results to 28 eligible papers. Further evaluation of the full-text papers led to elimination of another eight papers (including a review paper) because they did not provide relevant information on BFIs. The reference lists of the review paper [17] and of the selected full-text papers were examined to identify further relevant works. As a result of this selection process, 20 papers identified by the web search and six papers identified by the analysis of the reference list were included in our systematic review (Figs. 1 and 2). One additional study was identified through our secondary search for organosulfur compounds [18]. Among the selected papers, 13 describing excretion of quercetin and other flavonols after onion and shallot intake were excluded from the table of studies reporting candidate biomarkers (Table 1) due to the lack of specificity of these compounds. The resulting 14 articles included 10 human meal studies in which excretion of targeted organosulfur compounds have been analyzed following the ingestion of onion (1 study) or garlic (10 studies), one placebo-controlled intervention study, one cross-sectional study, one randomized, controlled parallel trial, and one randomized, controlled crossover intervention study. No observational studies on a larger (> 100) number of subjects were found. The studies reporting non-specific biomarkers are summarized in Additional file 1: Table S1 to provide all the relevant information collected during the literature search, and the reasons for inclusion or exclusion from Table 1 is summarized in Table 2.

*S*-allylmercapturic acid (ALMA) in urine, allyl methyl sulfide (AMS) in breath or urine, allyl methyl sulfoxide (AMSO) in urine, allyl methyl sulfone (AMSO_2_) in urine, and *S*-allyl cysteine (SAC) in blood/plasma were selected as candidate BFIs for garlic intake due to their specificity and suitable post-prandial kinetics and dose-response. No candidate BFIs were selected for onion intake as a result of the lack of specificity of quercetin. Urinary *N*-acetyl-*S*-(2-carboxypropyl)cysteine (CPMA) in urine was suggested as candidate BFIs for both onion and garlic intake, which may be promising biomarker for the assessment of the intake of the entire *Allium* vegetable group; however, further studies are needed to test this possibility. Potential BFIs for chives, leek, or other *Allium* species have not yet been suggested in the literature. The chemical description of these candidate BFIs is shown in Additional file 1: Table S1.

**Fig. 3:**
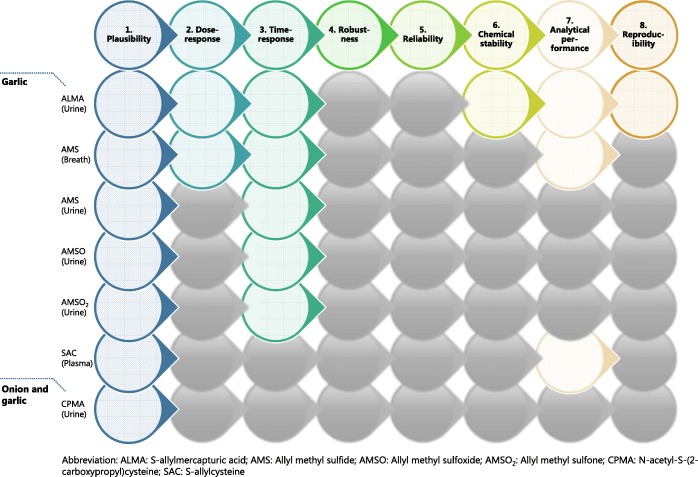
Overview of the validation process and its application for candidate BFIs for *Allium* vegetables. Colored circles refer to the answer “yes, the criterion is fulfilled for at least some use of the biomarker,” black circles refer to the answer “the criterion has been investigated but it was not fulfilled,” and gray circles refer to the answer “the criterion has not been investigated or data is not available”

A set of eight validation criteria according to a recent methodology [14] were applied on these four candidate BFIs, and the result is shown in Fig. 3. As a result, ALMA appears to be a promising BFI for garlic intake meeting six of the eight criteria. Adding information from observational studies on robustness, in comparison with best current practice (24 h recalls) and possibly also studies with repeated exposure to garlic to provide better information on kinetics, should help providing a qualitative-level biomarker to support information from dietary instruments such as food questionnaires or food diaries. Breath AMS met four out of the eight criteria but needs more extensive further validation. None of the other currently identified BFIs suffices for estimating onion, garlic, or total *Allium* intake.

## Discussion

*Allium* vegetable consumption has been investigated over the past 30 years, principally via targeted methods, due to the potential health benefits provided by specific components, such as flavonoids [9] or allicin [8]. Therefore, the majority of the available studies focus on these components.

### Garlic biomarkers

Several independent studies have identified *S*-allylmercapturic acid (ALMA), known also as *N*-acetyl-*S*-allyl-L-cysteine, as a biomarker of garlic intake. These studies also provide information about its urinary excretion profile [19–21]. ALMA is a metabolite of γ-glutamyl-*S*-allyl-L-cysteine (GAC), the primary sulfur compound found in the intact garlic. This compound is first hydrolyzed by γ-glutamine-transpeptidases to *S*-allyl-L-cysteine, and subsequently *N*-acetylated by *N*-acetyltransferases into ALMA to be excreted into urine, where it can be detected by GS-MS [20, 21]. De Rooij and his team [21] determined that the average elimination half-life of ALMA excretion was 6.0 ± 1.3 h, based on a test of five volunteers in a placebo-controlled intervention study indicating about 95% excretion within 24 h and suggesting that 24-h urine could be a reliable sample type in which to detect and quantify this compound [21, 22]. Verhagen and co-workers [22] successfully applied this methodology to check the compliance of garlic intake in a placebo-controlled intervention study. In addition, they used the method to check the intake of garlic recorded by dietary records in a cross-sectional study of 21 vegans versus 21 controls in Finland. In this latter study, no difference in garlic consumption or ALMA excretion was recorded between the two groups, and the ALMA levels were consistent with the results of the 5-day dietary records [22]. The number of subjects may still be seen as too low to validate the robustness of the biomarker, and more studies in different populations are needed to assess the actual specificity and robustness of ALMA as a BFI for garlic. In another study, Cope and co-workers [20] measured ALMA in urine to assess compliance to the consumption of different doses of garlic showing a positive correlation between its excretion and the dose consumed, even though the variability among individuals was high, depending on differences in metabolism by *N*-acetyltransferase isoforms [20, 21]. For controls and subjects who ingested up to 1 g garlic, ALMA levels in 24-h urine were very low or under the limit of detection, but increased considerably when the intake was 3 or 5 g [20]. The contribution of other *Allium* vegetables to ALMA in plasma or urine has not been investigated. One confounder to take into account is the ALMA excretion related to occupational exposure to allyl-halides (e.g., allylchloride), as shown by de Rooij et al. [23]. Since only few people are usually exposed to allyl-halides, urinary ALMA could be considered as a promising candidate BFI for garlic, but additional observational studies are needed to better assess the robustness of the biomarker. Garlic supplements are available on the market and could potentially confound garlic intake measured by any biomarker, including ALMA. No studies have so far investigated ALMA excretion after garlic supplement intakes. Regarding analytical aspects, the biomarker has proven to be stable in urine at − 20 °C for at least 3 months [21] and two different GC-MS quantification methods for ALMA have been developed [20, 21]. In the oldest method, the limit of detection was 100 ng/ml [21], while in the more recent one, ALMA was detectable at levels of 4 to 176 ng/ml [20]. The method proposed by De Rooij et al. [21] was also reproduced with slight modifications by Verhagen et al. [22].

Besides ALMA, other compounds also increase in urine after garlic consumption, namely urinary hexahydro-hippuric acid and *N*-acetyl-*S*-(2-carboxypropyl) cysteine (CPMA) [19]. The former compound has also been observed after the intake of berries [24], while the latter was reported by Jandke et al. in urine also after onion consumption [19], suggesting that this compound may be a general candidate BFI for the entire class of *Allium* vegetables. Further studies are needed to assess the specificity and robustness of CPMA.

Other compounds related to the exposure to fresh garlic are the allyl sulfides. These compounds give garlic its characteristic odor [8] and also derive, as ALMA, from γ-glutamyl-*S*-allyl-L-cysteine (GAC), which can be hydrolyzed and oxidized to yield *S*-allyl-L-cysteine sulfoxide (alliin). Alliin is subsequently transformed to allicin during chewing or cutting due to activation of the enzyme, alliinase. Allicin is highly unstable and instantly decomposes to form various lipid-soluble compounds including diallyl sulfide (DAS), diallyl disulfide (DADS), and diallyl trisulfide (DATS), while the main volatile metabolites, such as allyl methyl sulfide (AMS) and allyl methyl disulfide (AMDS), may be formed in vivo by the action of glutathione on DADS and DAS or on other components containing the C_3_H_5_-S-moiety [25]. It is worth noting that allyl sulfides can be produced only in the presence of alliinase, which is released by chopping, crushing, chewing, or blending garlic, causing maximal allicin production before reaching the intestinal tract. Lawson and Wang [28] showed that in processed garlic (microwaved-cooked and vacuum-dried), as well as in supplements, this enzyme does not have the opportunity to convert alliin to allicin. Compounds such as AMS were therefore not found in breath as when exposed to garlic powder tablets where allicin is added from the beginning. At the same time, GAC also converts to water-soluble organosulfur compounds including *S*-allyl cysteine (SAC) and *S*-allyl mercaptocysteine (SAMC). The former compound can be absorbed by the body and can be detected in plasma by HPLC-MS using atmospheric pressure chemical ionization (APCI)-MS, as shown by Rosen et al. [18]. The analytical method was described but kinetic information was lacking, and further studies are needed to characterize absorption and excretion of these compounds.

Bartzatt and his team [27] detected a series of the lipophilic sulfur compounds (DADS, DAS, and DMDS) derived from ingested garlic oils in urine by GC-MS. These metabolites have also been detected and monitored in volunteers’ breath after consumption of garlic, as well as other components such as allyl mercaptan, AMS, AMDS, DATS, and acetone [18, 28–32]. After garlic intake, AMS was the most abundant organosulfur compound in breath [18, 28, 30], followed by DAS [18] and DADS [30]. These metabolites demonstrated two different excretion profiles in breath. Taucher et al. [29] showed that AMDS, DAS, DADS, and DATS peaked shortly after ingestion of garlic and declined to baseline values within the next 2–3 h, while the concentration of breath AMS, DMS, and acetone increased much more slowly, showing elevated values even 30 h after garlic consumption. It has been suggested that the higher levels and longer presence of AMS in breath could be due to the fact that this compound is produced by the gut microbiota, while the short-term observations of the other compounds could result directly from formation in the mouth [31]. These authors also observed transient high concentrations of methanethiol, allyl mercaptan, and other allyl sulfides immediately after garlic ingestion, but these molecules disappeared after 2 h. Furthermore, Lawson and Wang [28] identified a linear dose-response relationship between AMS exhalation within 48 h and allicin consumption, which was equivalent to the intake of 7, 3.5, and 1.8 g of thoroughly crushed fresh garlic. Therefore, they proposed breath AMS as a suitable biomarker to assess garlic consumption, as it was absent from breath when garlic was not consumed. High inter-individual variation was observed (CV = 54%), while the variation for one person ranged between 12 and 20%. When 7 g of fresh garlic were consumed for three consecutive days, AMS levels did not show any accumulation proving that breath AMS expresses exposure to this food within the last 24 h. Regarding analytical validation, different studies proposed validated GC-MS methods in order to detect and quantify this compound in breath [18, 28, 30, 31]. AMS, together with its two oxidation products, allyl methyl sulfoxide (AMSO) and allyl methyl sulfone (AMSO_2_), were also observed in urine and breast milk [33, 34]. They were shown to peak around 1–4.5 h in urine after garlic consumption with distinct inter-individual variation, and the concentration of AMSO and AMSO_2_ were much higher than AMS. AMS and its oxidation products were detected in urine using GC-MS/O and HRGC-MS, respectively [33]. No information about the validation of these methods has been provided, and between-laboratory validation is missing. These results suggest that the specificity of AMS, AMSO, and AMSO_2_ in breath and urine makes it a promising biomarker for raw chewed or crushed garlic intake. Cooking processes may affect the excretion of AMS [28], and further studies should investigate whether this compound could only be used as a biomarker for raw garlic exposure. Other oil-soluble sulfur compounds, such as DADS, DAS, and DATS, have shown a very short-term presence in the body and, therefore, may not generally be suitable as garlic intake biomarkers.

Cai et al. [30] reported the identification and quantification of dimethyl selenide and other selenium-containing compounds in human breath by means of gas chromatography coupled to atomic emission detection (GC-AED). These molecules were in significantly lower concentrations than their sulfur analogs, making their quantification challenging. Furthermore, they did not seem to perform any better as BFIs for garlic compared to their sulfur analogs.

### Onion biomarkers

Onion is by far the richest dietary source of quercetin derivatives, with content exceeding 1.3 g/kg FW in some yellow varieties [35, 36]. The quercetin glucosides quercetin-4′-*O*-glucoside, quercetin-3,4′-*O*-diglucoside, and isorhamnetin-4′-*O*-glucoside are peculiar to onions and shallots and can be readily absorbed into the gut lining in the small intestine, in a more efficient way than quercetin itself or quercetin glycosides found in other food sources such as black tea, apples, and wine [37–39]. Therefore, these compounds, as well as their metabolites, can potentially be detected in body fluids shortly after ingestion and may represent potential short-term intake BFIs for recent onion consumption. However, studies on quercetin glucoside metabolism have not yet provided solid evidence for this. The quercetin glucosides present in onion are absorbed and modified in the body to produce the same quercetin metabolites as those observed after the consumption of any other quercetin glycoside or the aglycone; thus, they are common to many other quercetin-containing foods [26, 40–43]. During their absorption into the epithelial intestinal cells, the glucosides are hydrolyzed and the released quercetin is further glucuronidated, sulfated, and/or methylated by UDP-glucuronosyl transferases (UGTs), sulfotransferases (SULTs), and catechol-*O*-methyl transferase (COMT) in intestinal and hepatic cells. Mullen and co-workers [41] were able to identify methyl-, glucuronosyl-, and sulfo-conjugates, as well as glucosyl-conjugates, of quercetin both in plasma and urine by HPLC/MS-MS, providing a broad picture of the absorption and metabolism of quercetin-glucoside metabolites. The major conjugated metabolites detected in plasma were quercetin-3′-sulphate and quercetin-3-glucuronide, with a variety of minor components, such as quercetin glucuronide sulfates, quercetin diglucuronides, and isorhamnetin-3-glucuronide. The *t*_max_ in plasma for these compounds was below 1 h, except for quercetin glucuronide sulfate, which peaked at 2.5 h. The major urinary components were quercetin-3′*O*-glucuronide, two quercetin glucoside sulfates, and a methylquercetin diglucuronide. These metabolites may derive from further metabolism of quercetin-3′-sulphate and quercetin-3-glucuronide before returning to the bloodstream and being excreted in urine via the kidneys [41]. Extensive metabolism by the microbiota also occurs in the large intestine, which converts quercetin and its conjugated derivatives into small phenolic acids such as hydroxyphenylacetic acids and 4-hydroxyhippuric acid [44, 45]. Such results indicate that extensive modification of quercetin glucosides occurs following ingestion of onions. Hong and Mitchell [46], who identified 21 flavonol metabolites in human urine after the consumption of cooked onion, reported considerable differences in the levels of metabolites among individuals. They suggested that monitoring the range of quercetin metabolites as a biomarker for flavonol consumption may reveal information on inter-individual biotransformation capacity (e.g., a host factor), while failing as a method for general monitoring of onion intake or flavonoid-rich food intake. In addition, a quantification of the parent compounds producing these molecules is extremely complex. Finding of native isorhamnetin-4′-*O*-glucoside and of quercetin-4′-*O*-glucoside has been reported in plasma [47], but were subsequently shown to be caused by limitations in the ability of the analytical methodology to discriminate between glucosides and glucuronides [26, 41]. Due to the extensive metabolism of quercetin glucosides, the large inter-individual variability in the generated metabolite profiles in plasma or urine, and the wide distribution of other quercetin glycosides in many plant-based foods providing the same metabolites, these compounds cannot be considered as promising BFIs for onion.

More specific dietary biomarkers related to *Allium* vegetable consumption are organosulfur compounds, which have been investigated in garlic and onion due to their suspected antibacterial and anticancer activities [6]. In an older study, Jandke and Spiteller [19] observed *N*-acetyl-*S*-(2-carboxypropyl) cysteine (CPMA) and *S*-allylmercapturic acid (ALMA) in human urine after both onion and garlic consumption. CPMA was already present in low concentration in urine at baseline and increased dramatically after the ingestion of onion. This compound may be a possible metabolite of γ-glutamyl-*S*-(2-carboxypropyl)cysteinylglycine, a glutathione derivative present in onion. Only one paper reported detection of ALMA in urine after onion consumption, but no data are available regarding the analytical validation of the method, kinetics, and dose-response [19]. Subsequent studies on ALMA investigated exclusively garlic intake; therefore, it is uncertain whether it can be considered as a candidate biomarker of onion consumption as well. Posma and his team [48] identified *N*-acetyl-*S*-(1Z)-propenyl-cysteine-sulfoxide (NAcSPCSO) in urine after onion consumption. They suggested that this compound might be a metabolite of *S*-propenyl-cysteine-sulfoxide (SPCSO), which is the major flavor precursor in onion.

It is worth noting that one paper on an animal model reported an untargeted approach for the evaluation of metabolic effects associated with onion intake [49]. In this study, ^1^H-NMR spectroscopy was used to compare the urine metabolome of rats consuming normal food and rats fed with an onion diet. Two highly discriminant signals for onion intake were identified as dimethyl sulfone and 3-hydroxyphenylacetic acid. None of these compounds is sufficiently specific to assess onion intake. The former can be present in other plant-derived foods [50], used as food supplements [51], or can originate from human endogenous cysteine and methanethiol metabolism [52], while 3-hydroxyphenylacetic acid is mainly derived from gut microbial fermentation of polyphenols, such as quercetin, and hesperetin [44, 53], and therefore common with many other foods.

### Shallot biomarkers

Shallots have a similar flavonol composition as onion [36]. Only one paper was found on shallot intake; here, plasma quercetin was monitored after shallot intake [54]. It was shown how differences in the food matrix (shallot flesh or dry skin) could affect the plasma quercetin profile, and quercetin aglycone was found to be more bioavailable than its glucosides when provided along with dietary sources. No other investigation has been made to evaluate the presence of other specific metabolites associated with shallot intake although the food chemistry of the wild (Persian) shallot indicates that *N*-hydroxypyrithione sulfinates could be putative biomarkers related to intake of this species.

### *Allium* vegetable group

CPMA seems to have the potential to assess intake of *Allium* vegetables in general, but additional studies are needed to evaluate its usefulness as BFI for other *Allium* vegetables except for onion and garlic. No studies have attempted to use a combination of these biomarkers as a combined BFI for *Allium* vegetable intakes.

### Validation of candidate BFIs

From the validity evaluation of candidate BFIs for *Allium* vegetables (Fig. 3), ALMA may represent a good qualitative exposure biomarker for short-term intake of garlic, but its usefulness as quantitative biomarker to assess garlic intake has to be confirmed, as the levels of its precursor GAC is quite variable in garlic [55] and the conversion rate to *N*-acetyl-*S*-allylcysteine is highly variable due to possible differences in subject metabolism. Additional observational studies on robustness, studies with repeated exposure to garlic and comparison with other measures are needed to reach the full validation according to all criteria. AMS could be a biomarker for garlic intake when it is consumed raw with chewing or after crushing or cutting, e.g., in salads and dressings. The dose response and kinetics of AMS are well established in breath samples while only little information is available for urine samples. Further studies are needed to check its robustness, reliability, and stability in both sample types and to evaluate its applicability after intake of cooked garlic. AMSO and AMSO_2_ have been detected in urine with a well-defined time-response relationship but the high inter-individual variation might limit their use. SAC in plasma has been discovered and measured with a well-described method. However, information is lacking in all the other aspects of validation, and this candidate BFI therefore needs to be evaluated in further studies. Also it is still not clear whether ALMA, AMS, AMSO, and AMSO_2_ are solely related to garlic or, instead, to the whole food group. CPMA has been detected in urine after both garlic and onion intake indicating that it could be a promising biomarker for estimating intake of *Allium* vegetables in general. CPMA was found to be present also at baseline in urine. It has been investigated only in one study and there is not enough information regarding the validity of the analytical method. Therefore, its validity as a BFI needs to be further documented in controlled dietary interventions and observational studies.

## Conclusion

Several compounds have been found to increase in urine, blood, or breath after consumption of different *Allium* vegetables with potential as BFIs for the specific species or for the whole group. However, only a few compounds were selected here as candidate BFIs based on the evaluation of their specificity and concentration in human samples after intake. Five compounds candidate as BFIs for assessment of garlic consumption, ALMA, AMS, AMSO, AMSO_2_, and SAC, while no candidate BFIs were found to be specific for intake of any other *Allium* vegetables. The five biomarkers have been shown to be promising biomarkers for the assessment of recent garlic intake based on three or more validation criteria. However, further validation is needed, in particular since their sensitivity and specificity have never been assessed in observational studies. Further studies are needed to evaluate whether these biomarkers are solely related to garlic consumption or to the whole *Allium* group. CPMA derived from garlic as well as from onion components has been found in high levels in urine. This suggests that it may also represent a possible biomarker to assess intake of vegetables from the *Allium* food group. However, very little information is available from studies in humans on CPMA, and intervention trials as well as observational studies are therefore needed to assess its performance as a BFI. Untargeted analyses of human samples should be performed after controlled intakes of each of the common *Allium* vegetables, including garlic, onion, shallots, leek, chives, and ramsons, as well as after the intake of supplements containing dried, or otherwise processed, *Allium* products. Thus, further discoveries as well as further validation studies are needed in this area to identify reliable biomarkers reflecting *Allium* vegetable intake while discriminating between the consumption of individual *Allium* vegetables.

## Additional file

Additional file 1: **Table S1.** Chemical description of the candidate BFIs for *Allium* vegetables. Text S1 Validation criteria for biomarkers of food intake. (DOCX 20 kb)
