# Supplementary material for: Biomarkers of food intake for Allium vegetables
Source: Genes Nutr. 2018 Dec 27;13:34. doi: 10.1186/s12263-018-0624-4 (PMC6309086; doi:10.1186/s12263-018-0624-4)
Supplement: Supplementary file 1 — Table S1. Chemical description of the candidate BFIs for Allium vegetables. Text S1 Validation criteria for biomarkers of food intake. (DOCX 20 kb) [file 12263_2018_624_MOESM1_ESM.docx]

**Table S1.** Chemical description of the candidate BFIs for *Allium* vegetables*

| **Food item** | **Candidate BFI** | **Biofluid Locations** | **Chemical formula** | **Monoisotopic molecular weight** | **HMDB ID** |
| --- | --- | --- | --- | --- | --- |
| **Garlic** | ALMA | Urine | C8H13NO3S | 203.062 | NA |
|  | AMS | Breath/Urine | C4H8S | 88.035 | HMDB0031653 |
|  | AMSO | Urine | C4H8OS | 104.030 | HMDB0112254 |
|  | AMSO2 | Urine | C4H8O2S | 120.025 | HMDB0112255 |
|  | SAC | Plasma | C6H11NO2S | 161.051 | HMDB0034323 |
| **Onion and garlic** | CPMA | Urine | C9H15NO5S | 249.067 | NA |

* Information source: Human Metabolome Database (HMDB), Pubchem.

Abbreviation: ALMA: S-allylmercapturic acid; AMS: Allyl methyl sulfide; AMSO: Allyl methyl sulfoxide; AMSO2: Allyl methyl sulfone; CPMA: N-acetyl-S-(2-carboxypropyl)cysteine; SAC: S-allylcysteine; NA: Not available.

**Text S1.** Validation criteria for biomarkers of food intake according to Dragsted et al [14].

| **Validation criterion** | **Validation questions and their sub-criteria for answering “yes”** |
| --- | --- |
| 1. Plausibility | ***Q: Is the marker compound plausible as a specific BFI for the food or food group (chemical/biological plausibility)?***  ((The BFI is likely to be a metabolite or process-related derivative of a compound known to occur in the food or food group) OR (the BFI has been identified as a putative biomarker for the food/food group) OR (the compound was identified as a putative biomarker in a metabolomics investigation))  AND (variability of (parent) compound within food or food group (if known) is limited)  AND ((the level of the (parent) compound in other foods is comparatively low) OR (presence only in other foods not commonly consumed)) |
| 2. Dose-response | ***Q: Is there a dose-response relationship at relevant intake levels of the targeted food (quantitative aspect)?***  (The dose-response relationship of the BFI has been established using several intake levels (in a meal study) OR (in different meal studies where the results were comparable) OR (in cross-sectional study or longitudinal observational studies))  AND (the background level of the BFI is 0 or low)  Information about the limits for common background levels and saturation kinetics of the BFI should be provided as a comment |
| 3. Time-response | ***Q: Is the biomarker kinetics described adequately to make a wise choice of sample type, frequency and time window (time-response)?***   1. (The single-meal time-response relationship of the BFI has been described for a defined sample type and time window in a meal study) OR |
|  | 1. (The kinetics of the BFI after repeated intakes has been described for a defined sample type in a meal study) OR (accumulation of the BFI in certain sample types has been observed)   Information about ADME and enzyme induction, inhibition or altered excretion in the metabolism of the BFI or its precursor could be provided as a comment |
| 4. Robustness | ***Q: Has the marker been shown to be robust after intake of complex meals reflecting dietary habits of the targeted population (robustness)?***  ((The BFI has been measured and shown to be robust after intake of complex meals (in intervention studies) OR (in observational studies))  AND ((There is no confounding food observed) OR (The level of the BFI from the confounding food is low) OR (The confounding foods are not commonly consumed)) |
| 5. Reliability | ***Q: Has the marker been shown to compare well with other markers or questionnaire data for the same food/food group (reliability)?***  (The BFI has been compared well (with other biomarkers for the same food or food group) OR (with dietary assessment instruments) OR (with data in studies with highly controlled setting and supervised intake) |
| 6. Stability | ***Q: Is the marker chemically and biologically stable during biospecimen collection and storage, making measurements reliable and feasible (stability)?***  (The BFI is chemically and biologically stable during biospecimen collection, processing and storage) OR (The BFI is not stable but suitable protocol has been established to achieve the stabilization of the BFI) |
| 7. Analytical performance | ***Q: Are analytical variability (CV%), accuracy, sensitivity and specificity known as adequate for at least one reported analytical method (analytical performance)?***  (The protocol of the method has been well described and could be repeated)  AND (The method has been compared with validated method or references)  AND (The analytical variability (CV%), accuracy, sensitivity and specificity have been described) |
| 8. Reproducibility | ***Q: Has the analysis been successfully reproduced in another laboratory (reproducibility)?***  (The analysis with the same method has been performed in at least 2 different laboratories)  AND (The measurements of the BFI obtained from different laboratories are comparable) |
